# Self-similar conductance patterns in graphene Cantor-like structures

**DOI:** 10.1038/s41598-017-00611-z

**Published:** 2017-04-04

**Authors:** H. García-Cervantes, L. M. Gaggero-Sager, D. S. Díaz-Guerrero, O. Sotolongo-Costa, I. Rodríguez-Vargas

**Affiliations:** 1 Centro de Investigación en Ciencias, Instituto de Investigaciones en Ciencias Básicas y Aplicadas, Universidad Autónoma del Estado de Morelos, Av. Universidad 1001, Col Chamilpa, 62209 Cuernavaca Morelos Mexico; 2 CIICAp, IICBA, Universidad Autónoma del Estado de Morelos, Av. Universidad 1001, Col. Chamilpa, 62209 Cuernavaca, Morelos Mexico; 3 Unidad Académica de Física, Universidad Autónoma de Zacatecas, Calzada Solidaridad Esquina Con Paseo La Bufa S/N, 98060 Zacatecas, Zac. Mexico

## Abstract

Graphene has proven to be an ideal system for exotic transport phenomena. In this work, we report another exotic characteristic of the electron transport in graphene. Namely, we show that the linear-regime conductance can present self-similar patterns with well-defined scaling rules, once the graphene sheet is subjected to Cantor-like nanostructuring. As far as we know the mentioned system is one of the few in which a self-similar structure produces self-similar patterns on a physical property. These patterns are analysed quantitatively, by obtaining the scaling rules that underlie them. It is worth noting that the transport properties are an average of the dispersion channels, which makes the existence of scale factors quite surprising. In addition, that self-similarity be manifested in the conductance opens an excellent opportunity to test this fundamental property experimentally.

## Introduction

Graphene is called to be a revolutionary material due to its outstanding physical properties and its technological potentiality^1–3^. From a fundamental perspective graphene also represents a laboratory at hand, since a multitude of exotic phenomena can now be a reality. Among the most remarkable effects reported to date we find Klein tunnelling^4, 5^, atomic collapse^6^ and the Hofstadter-Butterfly^7, 8^. The experimental verification of these phenomena has been possible thanks to the relativistic nature of the charge carriers in graphene, and to the experimental conditions required to observe them which are totally affordable in graphene. It is also important to mention that practically all mentioned phenomena have been proven through transport experiments. Transport measurements seem to be natural in graphene, in part, due to the two-dimensional nature of the material as well as its high transparency. Here, we report that the physical properties of a Cantor-like graphene-based structure can reflect the geometrical characteristics of the system. In particular, we show that the linear-regime conductance presents self-similar patterns with well-defined scaling rules, and even more, that these rules are directly related to the scale factors involved in the construction of the structure. As far as we know, our system is one of the few in which its geometrical characteristics are manifested directly in a measurable physical quantity. And even more, if our results are corroborated experimentally, a long quest to show that measurable physical quantities can express the geometrical characteristics of a system will finally be a reality. In solid state physics the mentioned quest start with the birth of quantum heterostructures. Specifically, since the pioneering work of Merlin *et al*.^9^ in aperiodic or quasi-periodic semiconductor superlattices it is well documented that physical properties such as the electronic and phononic spectra, the reflectance and transmittance, and even the wave function manifest fractal, self-similar and critical characteristics^10^, respectively. Particularly, self-similarity in most cases is considered as matter of visual perception and in rare occasions the scale factors that govern the self-similar patterns are given^11–13^. However, when we talk about self-similarity we are talking about scale factors, which connect the self-similar patterns at different scales. In short, self-similarity requires scale factors, in the same sense that fractality requires that the Hausdorff-Besicovich dimension be greater than the topological one^14^. Within this context, self-similarity in aperiodic structures in graphene is not the exception. Several works claim that the transmission probability sustains self-similar characteristics in Fibonacci and Thue-Morse superlattices^15–18^, but once again the scale factors that connect the transmission patterns are not derived. Recently, we have shown that the transmission properties of graphene subjected to self-similar and self-affine multi-barrier structures present self-similar characteristics^19–21^. Even more, we have determined the scale factors that connect the transmission patterns. In addition, we found that the structures with better scaling in the transmission patterns are those in which the barriers are generated by substrates that open a bandgap in the electronic structure of graphene. In other words, structures in which the pseudo-spin in graphene is not conserved, and consequently Klein tunnelling is not present. In this work we have taken a step further showing that the transport properties in graphene under the influence of a self-similar (Cantor-like) multi-barrier structure present well defined scaling rules, and hence self-similarity. In particular, we were able to derive mathematical expressions that connect the conductance patterns. We also hope that our results be encouraging for experimentalists, since conductance is a physical quantity that nowadays is measured in practically all graphene laboratories.

## Results

### Theoretical framework

As we already mentioned, in previous works we have shown that graphene under appropriate nanostructuring can present self-similar characteristics^19–21^. To be specific, the transmission probability or transmittance sustains self-similar patterns with well defined rules. As far as we know the nanostructuring that gives rise to these peculiar and exotic propagation properties is the one that combines regions that correspond to massless and massive Dirac electrons. At this point, it is also important to mention that here we deal with the same structure studied in ref. 21. To this respect, and even though the mentioned system and the methodology to deal with it are well presented in ref. 21, we consider important to describe here the generals of the structure, and the fundamentals of the theoretical framework, mainly those related to the transport properties.

**Figure 1:**
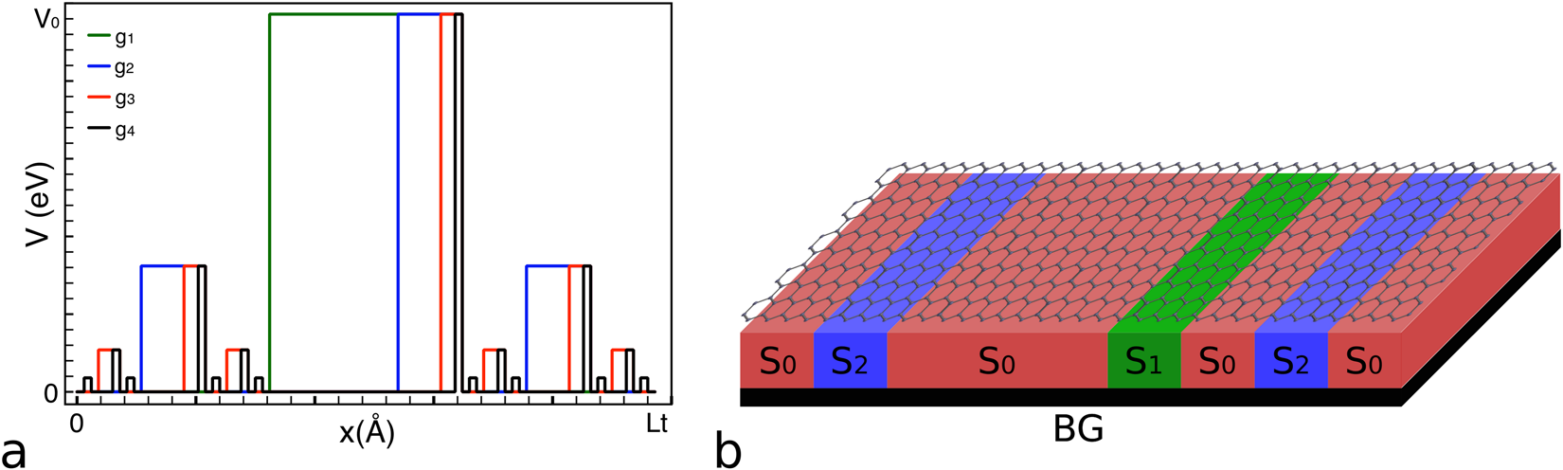
Self-similar multi-barrier structure^21^. (**a**) Potential or conduction band-edge profile of a Cantor-like multibarrier system. The first four generations are shown: green, blue, red and black lines, respectively. For this structure both coordinates, energy and distance, are scaled in Cantor-like fashion. (**b**) Schematic representation of the possible graphene Cantor-like structure. The graphene sheet is sitted or deposited on a nanostructured substrate. Here *S*_0_, *S*_1_, *S*_2_ etc. represent materials with different degree of interaction with the graphene sheet, such that, the different heights of the barriers required to get the Cantor-like structure be met. In particular, we are depicting the second generation of the self-similar multibarrier system.

Firstly, we describe how the potential or more precisely the conduction band-edge profile of our multibarrier structure is constructed. The construction of the self-similar multibarrier potential is divided in generations as follows: given an a priori set total length *Lt*, in the first generation (denoted by *g*_1_) *Lt* is divided in three parts of equal length (*Lt*/3). One rectangular barrier of height *V*_0_ is placed in the middle third, so the first generation consists of one barrier, called main barrier, of width *L*_0_ = *Lt*/3. Now, for the second generation (*g*_2_), we begin with *g*_1_. In each empty space at both sides of the barrier the process of generation 1 is repeated, i.e., each one is divided in thirds and in the middle ones is placed a barrier of height *V*_1_ = *V*_0_/3. Additionally, the main barrier is scaled in its width and placed in the right third of the original one. For further generations *g*_*i*_ the above process is iterated beginning with *g*_2_, as shown in Fig. 1a. This potential or conduction band-edge profile could be obtained by nanostructuring the substrate in which the graphene sheet is sitted or deposited on, see Fig. 1b. In particular, the substrate has to be nanostructured with materials with different degree of interaction with the graphene sheet, or in other words, with materials that induced different bandgap openings on graphene. In Fig. 1b these materials are represented by *S*_0_, *S*_1_, *S*_2_, etc. As far as we know, materials such as SiO_2_, SiC, hBN, etc. could be used for this purpose^22–24^.

From a theoretical standpoint graphene under the influence of a substrate, that opens a bandgap in the electronic structure, can be studied by means of the massive Dirac-like equation:

1 $$[{v}_{F}(\vec{\sigma }\cdot \vec{p})+t^{\prime} {\sigma }_{z}]\psi =E\psi ,$$

where $\vec{\sigma }=({\sigma }_{x},{\sigma }_{y})$, $\vec{p}=-i\hslash \nabla$, *σ*_*i*_ with *i* = *x*, *y*, *z* is the *i*th Pauli matrix, ${v}_{F}=3ta/2\hslash$ is the Fermi velocity, with *t* the nearest neighbor hopping energy, *a* the carbon-carbon distance (1.42 Å) and *t*′ = *E*_*g*_/2 the height of the barrier. This equation can be solved straightforwardly, giving a parabolic dispersion relation:

2 $${E}^{2}=\pm {\hslash }^{2}{v}_{F}^{2}({q}_{x}^{2}+{q}_{y}^{2})+{t}^{^{\prime} 2},$$

the corresponding wave function comes as:

3 $${\psi }_{\pm }=\frac{1}{\sqrt{2}}(\begin{array}{c}1\\ {v}_{\pm }\end{array}){e}^{\pm i{q}_{x}x+i{q}_{y}y},$$

where the coefficients *v*_±_ are given by:

4 $${v}_{\pm }=\frac{E-t^{\prime} }{\hslash {v}_{F}(\pm {q}_{x}-i{q}_{y})}.$$

here $\vec{q}$ represents the two-dimensional wavevector of massive Dirac electrons, which is determined by eq. (2).

The graphene’s dispersion relation is practically unaffected by the influence of substrates such as SiO_2_. So, mathematically the massless regions in our system can be described by the following Dirac-like equation,

5 $${v}_{F}(\vec{\sigma }\cdot \vec{p})\psi =E\psi ,$$

with the dispersion relation:

6 $$E=\pm \hslash {v}_{F}k,$$

and the wave function:

7 $${\psi }_{\pm }=\frac{1}{\sqrt{2}}(\begin{array}{c}1\\ {u}_{\pm }\end{array}){e}^{\pm i{k}_{x}x+i{k}_{y}y},$$

with

8 $${u}_{\pm }=\frac{E}{\hslash {v}_{F}(\pm {k}_{x}-i{k}_{y})}.$$

here $\vec{k}$ is the two-dimensional wave vector of the massless regions. The components of $\vec{k}$, *k*_*x*_ and *k*_*y*_, are determined by eq. (6) as well as the angle *θ* between them, tan *θ* = *k*_*y*_/*k*_*x*_.

Once we have well characterised the different regions that constitute the multibarrier structure, that is, that we know the dispersion relations, the wave vectors and the wave functions of all regions, we can compute straightforwardly the transmission probability and the linear-regime conductance with the well-known transfer matrix method^25, 26^. In this case, we consider a multi-barrier graphene system as the one shown in Fig. 1. This method allows us to establish a relationship between the incident wave, before the multibarrier structure, and the transmitted wave, after the multibarrier structure. In each interface, that form part of the structure, the continuity condition for the wavefunction must be met along the propagation direction (*x*-coordinate). The mentioned relationship is given between the incident wave coefficients *A*_0_ and *B*_0_, and the transmitted wave ones *A*_*N*+1_ and B_*N*+1_ = 0. Moreover, taking into account the conservation of the transversal momentum, *q*_*y*_ = *k*_*y*_, the following relationship can be established:

9 $$(\begin{array}{c}{A}_{0}\\ {B}_{0}\end{array})=M(\begin{array}{c}{A}_{N+1}\\ 0\end{array}),$$

where the transfer matrix is given by:

10 $$M={D}_{0}^{-1}[\prod _{j=1}^{N}({D}_{j}{P}_{j}{D}_{j}^{-1})]{D}_{0},$$

with the dynamic and propagation matrices:

11 $${D}_{0}=(\begin{array}{cc}1 & 1\\ {u}_{+} & {u}_{-}\end{array}),\,{D}_{j}=(\begin{array}{cc}1 & 1\\ {v}_{+j} & {v}_{-j}\end{array})\,{\rm{and}}\,{P}_{j}=(\begin{array}{cc}{e}^{-i{q}_{xj}{d}_{j}} & 0\\ 0 & {e}^{i{q}_{xj}{d}_{j}}\end{array}),$$

where $j=1,2,\ldots N$. *q*_*xj*_ and *d*_*j*_ represent the *x* wave vector component and the width of the j-th region. Actually, for those regions without barrier the wave vectors and the dynamic matrices will be *q*_*xj*_ = *k*_*x*_ and *D*_*j*_ = *D*_0_, respectively.

Knowing the transfer matrix we can readily calculate the transmission probability^27^;

12 $$T(E,\theta )=\frac{1}{{|{M}_{11}|}^{2}},$$

with *M*_11_ the element (1, 1) of the transfer matrix.

Without loss of generality, the transmission probability allows us to calculate the linear-regime conductance directly through the Landauer-Büttiker formalism^28^. Within this formalism the conductance for monolayer graphene can be written as,

13 $${\mathbb{G}}=\frac{G}{{G}_{0}}={E}_{F}^{\ast }{\int }_{-\frac{\pi }{2}}^{\frac{\pi }{2}}T({E}_{F}^{\ast },\theta )\cos \,\theta d\theta ,$$

where ${E}_{F}^{\ast }=\frac{{E}_{F}}{{E}_{0}}$ is the Fermi energy normalized to the height of the barrier *E*_0_ = *V*_0_ = *t*′, ${G}_{0}=\frac{2{e}^{2}{L}_{y}{E}_{0}}{{\hslash }^{2}{v}_{F}}$ is the conductance fundamental factor, L_*y*_ is the width of the system in the transversal direction *y*, and *θ* is the angle of incidence of the electrons with respect to the propagation direction *x*.

### Conductance scaling rules

The main goal of this work is to investigate the existence of scaling rules, and hence self-similarity, in the conductance patterns. For this, we follow the guidelines reported in our previous works^19–21^. Specifically, we explore the three scaling rules reported for the transmittance: 1) between generations, 2) between different heights of the main barrier, and 3) between different lengths of the structure. At this stage, it is important to mention that the scaling rules found for the transmittance cannot be implemented directly in the conductance, in part, because the conductance, for a given Fermi energy, is the result of the sum over all the transmission channels. As we will see in short in this case of the conductance the scaling rules are more intricate. Fortunately, as we will comment in the discussion section and we will present in the **supplementary information**, it is possible to define a quantity related to the conductance for which is much easier to visualise the scaling.

**Figure 2:**
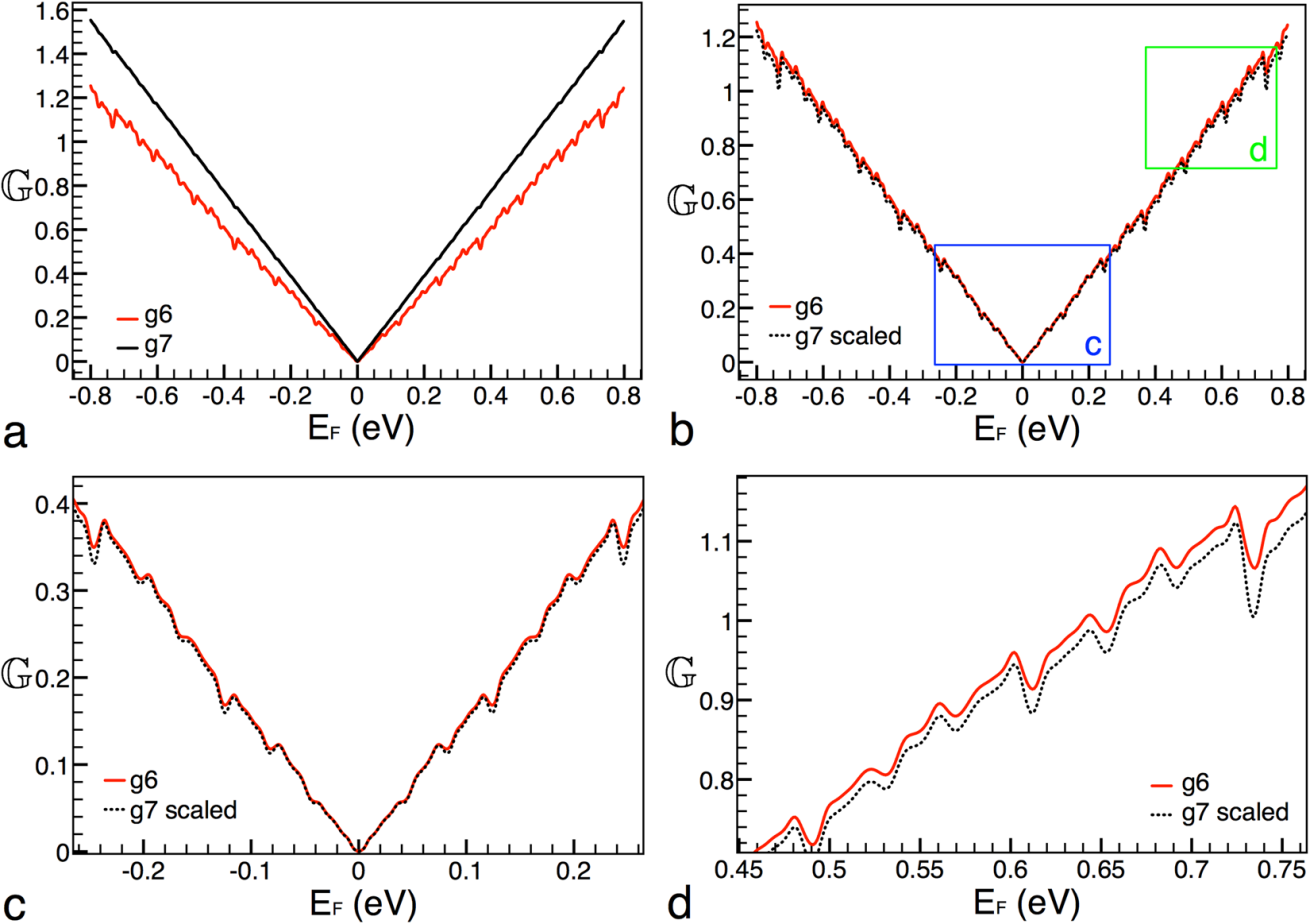
Scaling between generations. (**a**) Conductance patterns for generations 6 and 7, solid-red and solid-black lines, respectively. (**b**) The same as in (**a**), but in this case generation 7 is scaled according to eq. (14), dotted-black curve. The blue and green rectangles correspond to the zooms shown in (**c**,**d**). In these zooms we can appreciate the ripple structure of the conductance. The other parameters of the Cantor-like structures are: *V*_0_ = 0.2 eV and *Lt* = 10000 Å.

In first place, we want to see if there are signs of self-similarity between generations. Then, we have considered two generations, in specific, the sixth and seventh generation, while the height of the barrier and width of the structure remain fixed at *V*_0_ = 0.2 eV and Lt = 10000 Å, respectively. In Fig. 2a the conductance patterns for these generations are shown. As we can notice the conductance curves are dominated by the linear term of the Fermi energy with small ripples (better appreciated in Fig. 2c,d) caused by the angular average of the transmission channels, consistent with eq. (13). At first glance, it seems that there is not resemblance between the conductance curves. For instance, in the case of the curve that corresponds to the seventh generation the ripples are barely perceptible. However, with an appropriate transformation we can connect these generations, see Fig. 2b. In this case the transformation comes as ${{\mathbb{G}}}_{6}({E}_{F}^{\ast })=\frac{{[{{\mathbb{G}}}_{7}({E}_{F}^{\ast })]}^{9}}{{\mathrm{(2}{E}_{F}^{\ast })}^{8}}$, where the subscript is the generation number. It is interesting to note that a factor of 9 be involved in the transformation. In fact, this factor is the product of the two scale factors in the construction. That is, the scale factor for the energy times the scale factor for the length, i.e., (3)(3) = 9. Other important characteristic and difference with respect to the scaling found for the transmittance is the factor ${\mathrm{(2}{E}_{F}^{\ast })}^{8}$. Indeed, this factor is what really makes difficult to visualize the scaling rules in the conductance. In addition, it is possible to extend this scaling rule to two non consecutive generations,

14 $${{\mathbb{G}}}_{g}({E}_{F}^{\ast })\approx \frac{{[{{\mathbb{G}}}_{g+m}({E}_{F}^{\ast })]}^{{9}^{m}}}{{\mathrm{(2}{E}_{F}^{\ast })}^{{9}^{m}-1}},$$

where *m* is the difference between generations. This expression indicates that generations are connected by a power of the form 9^*m*^, that is, that somehow the power factors are accumulated in multiplicative fashion.

**Figure 3:**
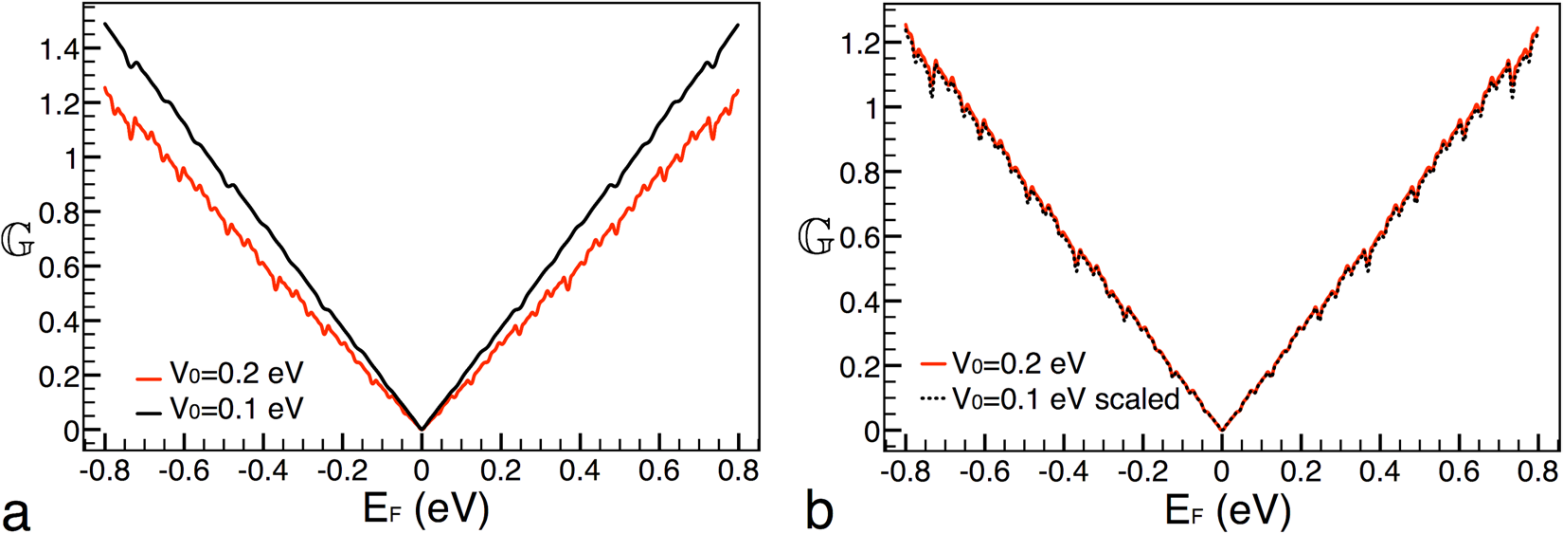
Scaling between different heights of the main barrier. (**a**) Conductance patterns for heights *V*_0_ = 0.2 eV and *V*_0_ = 0.1 eV, solid-red and solid-black lines, respectively. (**b**) The same as in (**a**), but in this case the curve that corresponds to *V*_0_ = 0.1 eV is scaled according to eq. (15), dotted-black curve. The other parameters of the Cantor-like structures are: *g*6 and *Lt* = 10000 Å.

Now it is turn to see the conductance scaling between different heights of the main barrier. In Fig. 3a the conductance patterns for this case are presented. The solid-red and solid-black curves correspond to *V*_0_ = 0.2 eV and *V*_0_ = 0.1 eV, respectively. The generation and length considered were *g*6 and *Lt* = 10000 Å. As we can appreciate the conductance patterns are quite similar to those between generations. However, in this case the scaling is totally different. In specific, the transformation comes as ${{\mathbb{G}}}_{0.2}({E}_{F}^{\ast })\approx \frac{{[{{\mathbb{G}}}_{0.1}(\frac{{E}_{F}^{\ast }}{2})]}^{4}}{\mathrm{2(2}{E}_{F}^{\ast }{)}^{3}}$, where the subscript indicates the height of the main barrier. So, the conductance needs to be risen to the fourth power. This power factor is related to the factor that connects the heights of the main barrier. Indeed, it is the same power factor previously found for the transmittance^21^. In addition, the conductance needs to be divided by $\mathrm{2(2}{E}_{F}^{\ast }{)}^{3}$ as well as the argument of it (the Fermi energy) reduced by a factor of 2. These two operations are also related to the factor between the heights of the main barrier. In Fig. 3b the result of the mentioned transformation is presented. As we can see the target curve (solid-red) and the scaled one (dotted-black) match pretty well. Furthermore, the transformation can be extended to two arbitrary barrier heights,

15 $${{\mathbb{G}}}_{{V}_{0}}({E}_{F}^{\ast })\approx \frac{{[{{\mathbb{G}}}_{\tfrac{1}{\kappa }{V}_{0}}(\frac{{E}_{F}^{\ast }}{\kappa })]}^{{\kappa }^{2}}}{\kappa {\mathrm{(2}{E}_{F}^{\ast })}^{{\kappa }^{2}-1}},$$

where *k* is the factor that connects the heights of the main barrier.

**Figure 4:**
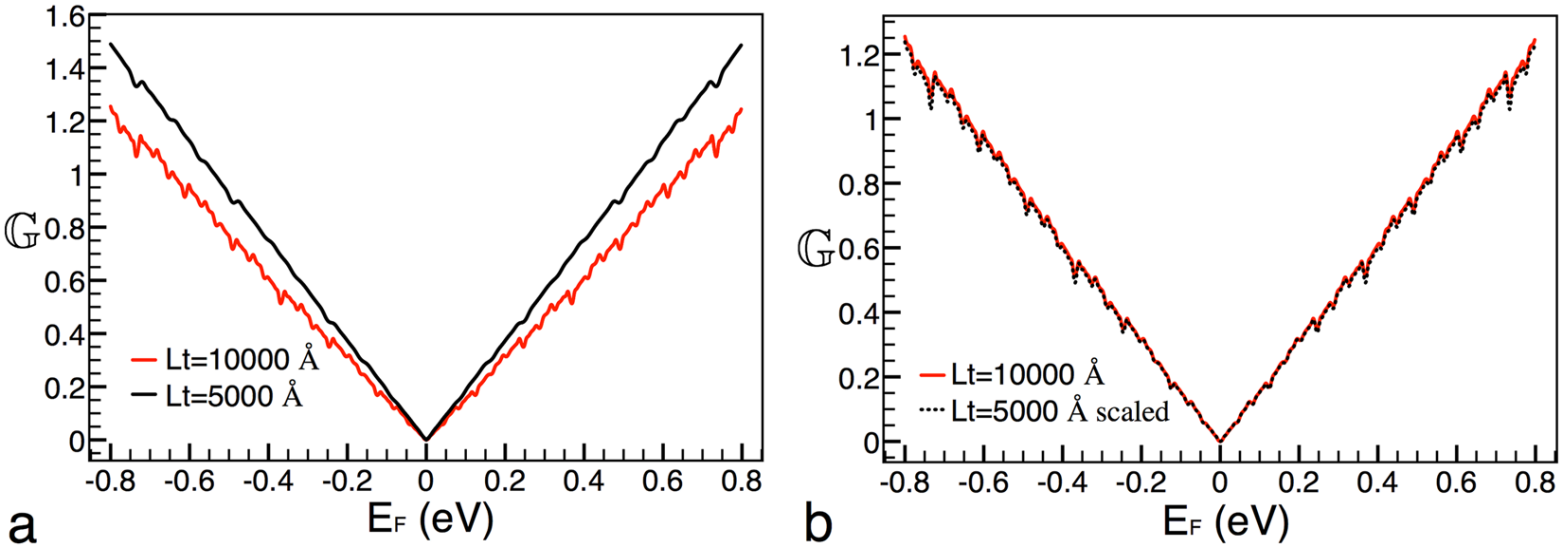
Scaling between different lengths of the system. (**a**) Conductance patterns for lengths *Lt* = 10000 Å and *Lt* = 5000 Å, solid-red and solid-black lines, respectively. (**b**) The same as in (**a**), but in this case the curve that corresponds to *Lt* = 5000 Å is scaled according to eq. (16), dotted-black curve. The other parameters of the Cantor-like structures are: *g*6 and *V*_0_ = 0.2 eV.

Now, we consider conductance patterns between different lengths of the system, see Fig. 4. Taking into account that we are changing a structural factor, and basically in the same proportion as in the case of the heights of the main barrier, is that we will try out the same transformation that in the preceding case: ${{\mathbb{G}}}_{10000}({E}_{F}^{\ast })\approx \frac{{[{{\mathbb{G}}}_{5000}(\frac{{E}_{F}^{\ast }}{2})]}^{4}}{\mathrm{2(2}{E}_{F}^{\ast }{)}^{3}}$, where the subscript indicates the length of the system. In this case the transformation works quite well too, see Fig. 4b. In general, the expression can be written as,

16 $${{\mathbb{G}}}_{Lt}({E}_{F}^{\ast })\approx \frac{{[{{\mathbb{G}}}_{\tfrac{1}{\alpha }Lt}(\frac{{E}_{F}^{\ast }}{\alpha })]}^{{\alpha }^{2}}}{\alpha {\mathrm{(2}{E}_{F}^{\ast })}^{{\alpha }^{2}-1}},$$

where *α* is the factor that connects the lengths of the system.

Last but not least, is that the individual transformations above presented, eqs (14), (15) and (16), can be combined to get a general expression,

17 $${{\mathbb{G}}}_{(g,{V}_{0},Lt)}({E}_{F}^{\ast })\approx \frac{{[{{\mathbb{G}}}_{(g+m,\tfrac{1}{k}{V}_{0},\tfrac{1}{\alpha }Lt)}(\frac{1}{\kappa \cdot \alpha }{E}_{F}^{\ast })]}^{{\mathrm{(9}}^{m}\cdot {k}^{2}\cdot {\alpha }^{2})}}{(\alpha \kappa \mathrm{)[2}{E}_{F}^{\ast }{]}^{{\mathrm{(9}}^{m}\cdot {k}^{2}\cdot {\alpha }^{2})-1}}.$$

**Figure 5:**
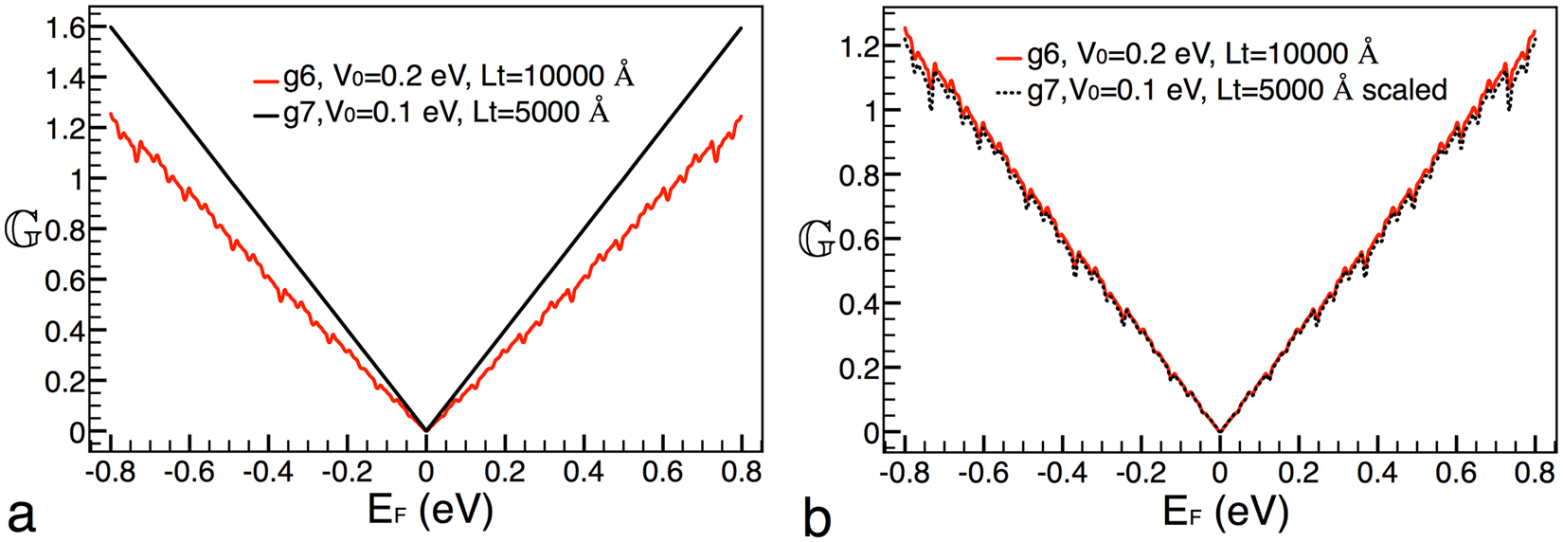
General scaling: combination of the individual scaling rules. (**a**) Conductance patterns for Cantor-like structures with parameters *g*6, *V*_0_ = 0.2 eV and *Lt* = 10000 Å and *g*7, *V*_0_ = 0.1 eV and *Lt* = 5000 Å, solid-red and solid-black lines, respectively. (**b**) The same as in (**a**), but in this case the solid-black curve is scaled according to eq. (17), dotted-black curve.

In order to see if this expression works, we have considered two Cantor-like structures with different generations, heights of the main barrier and lengths, see Fig. 5. In particular, the parameters of our reference structure are *g*6, *V*_0_ = 0.2 eV and *Lt* = 10000 Å, solid-red curve, while the parameters of the curve that will be scaled are *g*7, *V*_0_ = 0.1 eV and *Lt* = 5000 Å, solid-black curve, Fig. 5a. The specific transformation for our case is ${{\mathbb{G}}}_{\mathrm{(6},0.2,10000)}({E}_{F}^{\ast })\approx \frac{{[{{\mathbb{G}}}_{\mathrm{(7},0.1,5000)}(\frac{{E}_{F}^{\ast }}{2\cdot 2})]}^{\mathrm{(9}\cdot 4\cdot \mathrm{4)}}}{(2\cdot 2){[2{E}_{F}^{\ast }]}^{\mathrm{(9}\cdot 4\cdot \mathrm{4)}-1}}$. In Fig. 5b the results of this transformation are presented. As we can see the reference curve and the scaled one match quite well. This result is quite interesting, because, in principle, there is no warranty that the combination of the scaling rules would work.

**Figure 6:**
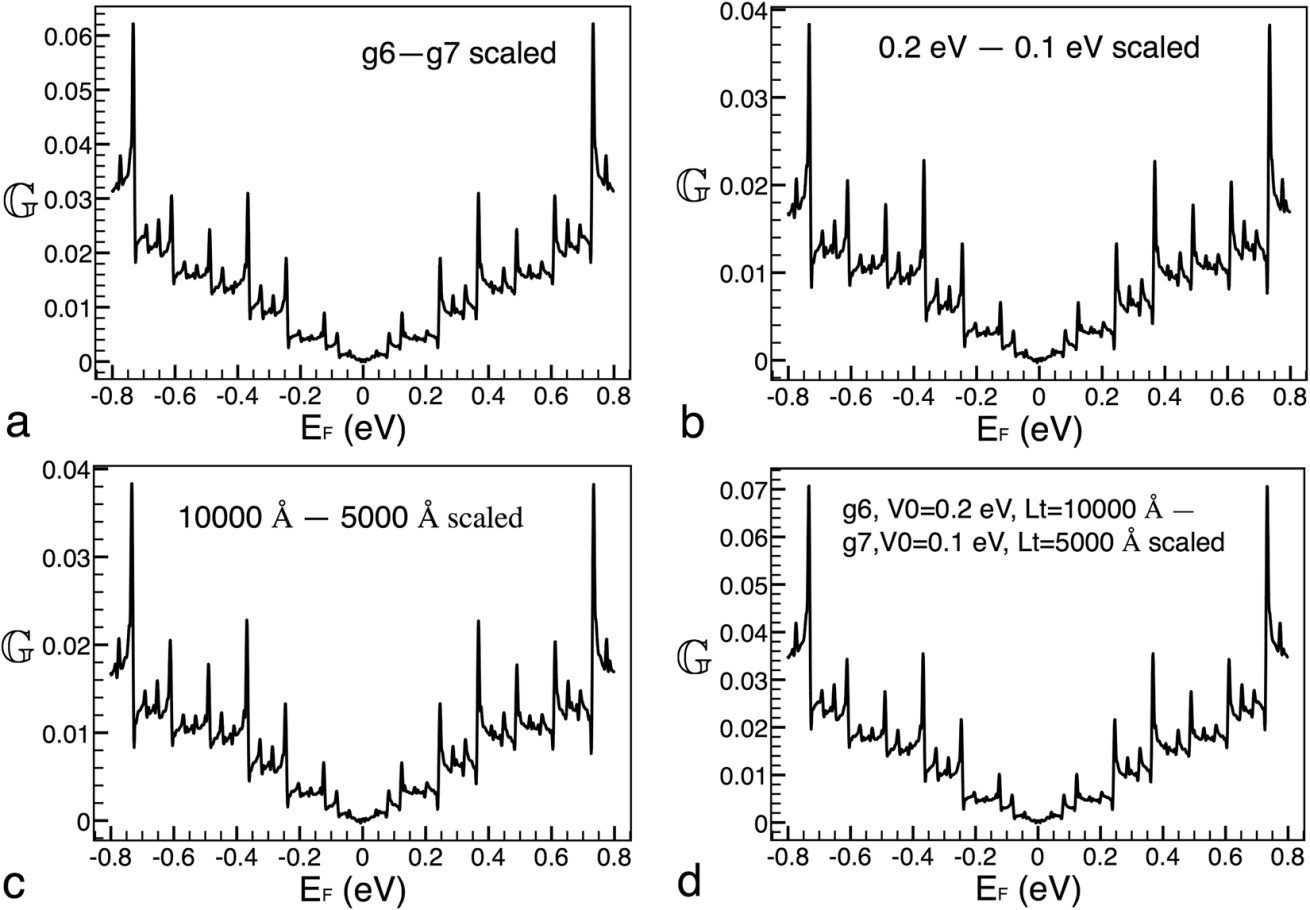
Difference between non-scaled and scaled curves for (**a**) generations, (**b**) heights of barrier, (**c**) lengths of the system and (**d**) the combination (general scaling) of the preceding ones. The non-scaled and scaled curves correspond to the results presented in Figs 2, 3, 4 and 5.

In order to know how good are the above presented scaling rules we have subtracted the non-scaled and scale curves of the conductance. The results of this subtraction are shown in Fig. 6. As we can see there are two main features in these results: 1) the matching is worsen as the Fermi energy rises and 2) there are a series of peaks at specific energies. These peaks are bigger as the Fermi energy increases and the localization corresponds mainly to minimums in the conductance. We can also notice that the matching for heights of the main barrier and lengths of the system is better than the corresponding one to generations, compare the vertical scale in Fig. 6a–c. The bigger difference between the non-scaled and scale curves is presented for the general scaling Fig. 6d. Another quantity that can help us to quantify the matching between the non-scaled and scaled curves is the root-mean-square-deviation (rmsd). The specific values of the rmsd for generations, heights of the main barrier, lengths of the system and the general scaling are 0.017274226, 0.01056703, 0.011058418 and 0.018950994, respectively. It is worth mentioning that even when we are dealing with a double average property the rmsds are acceptable and even better than the corresponding ones previously reported for the transmittance^20^.

## Discussion

In first place, we want to discuss the conditions under which the conductance scaling arises. According to our results, it is necessary that we have a self-similar structure, Dirac electrons (graphene) and that Klein tunneling be absent (non conservation of the pseudo-spin) in order to obtain self-similar characteristics in the conductance. In fact, these are the same conditions that have to be fulfilled to get scaling rules (self-similarity) in the transmission probability^19–21^. However, in the case of the conductance, we notice that to obtain scaling rules that be directly related to the scale factors involved in the construction of the structure, it is quite important that we scale the energy axis and the spatial coordinate (self-similar transformation) of the conduction band-edge profile in triadic-like fashion. In systems in which only the spatial coordinate or the energy axis are scaled (self-affine transformation) this connection between the scaling rules and the scale factors of the structure is not fulfilled, results that will be published elsewhere. This contrasts with the results obtained for the transmission probability, since in that case it is not necessary to scale both axis^20^. Other important characteristic of the system under study is that the scaling rules for the transmittance are also valid for oblique incidence, like in the case of simple Cantor-like structures^20^. Even more striking is that we can find scaling rules for the angular distribution of the transmittance, that is, the transmittance as a function of the angle for fixed energy, *T* = *T*(*θ*). As far as we have studied this characteristic is not at all present in other Cantor-like structures. It seem once again that the simultaneous scaling of both axis, energy and distance, is fundamental to get self-similarity in the physical properties. For more details see the corresponding section in the **supplementary information**.

**Figure 7:**
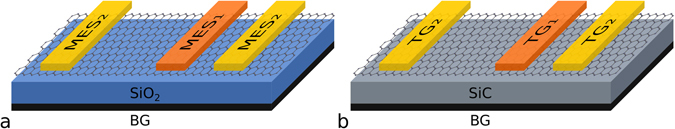
The same as in Fig. 1b, but here the proposed mechanisms to generate the barriers are (**a**) magnetic and electric fields and (**b**) a substrate with significant interaction with the graphene sheet in conjunction with an electric field. In (**a**) the graphene sheet is sitted or deposited on a non-interacting substrate like SiO_2_. In addition, top magnetoelectric strips are incorporated to modulate the distribution and shape of the magnetic and electric fields applied perpendicularly to graphene. A possibility for the magnetoelectric barriers is that the magnetic field be modulated in delta-like fashion and that the electrostatic field be arranged in stepwise fashion^31^. In (**b**) the graphene is deposited on a substrate with significant interaction like SiC. Besides, top gates are incorporated to create and modulate the so called electrostatic barriers^32^. Here, it is important to mention that with the incorporation of the magnetic field in (**a**) Klein tunneling is destroyed and with the SiC substrate along the graphene sheet in (**b**) the contrast of massless and massive regions is eliminated as well.

In second place, we consider that it is important to talk about the structure that we are proposing to obtain self-similar characteristics in the conductance. The mentioned structure is a challenge for experimentalists, not because a nanostructured substrate be a problem with the current technology, but because a substrate with regions with different degree of interaction with the graphene sheet, such that fulfill with the requirements of our system, is what is really difficult. To this respect, we believe that other options could be a possibility. For instance, a system with magnetoelectric barriers, barriers generated by magnetic and electric fields, is quite appealing, see Fig. 7a. In this case a nanostructured substrate is not necessary, instead top gates in which magnetic and electric field can be applied are needed. One of the main features of this system is that with the incorporation of the magnetic field the graphene’s pseudo-spin is not longer conserved, Klein tunneling not present. In this sense, this system is equivalent to ours, then we expect that the transmittance and conductance patterns show self-similar characteristics with well defined scaling rules. Other possibility, even more realistic, is the one depicted in Fig. 7b. In this case the graphene sheet is deposited on an interacting substrate such as SiC, then top gates are attached to apply an electric field. The substrate opens an energy bandgap in the entire structure and the electric field applied on the gates displaces the band structure generating the potential barriers. The presence of the bandgap eliminates the Klein tunneling and makes that electrons be of massive character in all region of the structure. Then, in principle, we can expect self-similar characteristic in the transmittance and conductance. Likewise, with this structure we can discern if the contrast between massless and massive regions in our systems is preponderant in the determination of the self-similar characteristics of the physical properties.

In third place, we want to argue about the expressions that connect the conductance patterns. In particular, we want to stress that the factors that appear in the denominator of the mentioned expressions are not easy to visualize. Indeed, at first instance, these factors make really difficult to obtain the scaling rules. At second instance, we can use an alternative quantity $H=H({E}_{F}^{\ast })$ in order to obtain straightforwardly the scaling rules. This quantity is directly related to the conductance, specifically $H({E}_{F}^{\ast })/{H}_{0}=G({E}_{F}^{\ast })/\mathrm{(2}{E}_{F}^{\ast }{G}_{0})$, or equivalently *H* is the angular average of the transmittance taken on half of the Fermi surface. One of the main characteristics of *H* is that is bounded, 0 ≤ *H* ≤ 1. This allows us to take *H* in a probabilistic sense, and most importantly, as first attempt, we can try to connect the *H* patterns with the transmittance scaling rules. In fact, the *H* scaling rules for generations and lengths of the system are the same as in the case of the transmittance at oblique incidence. However, in the case of the *H* scaling for different heights of the main barrier a contraction of the Fermi energy is required. For more details see the corresponding section in the **supplementary material.**

**Figure 8:**
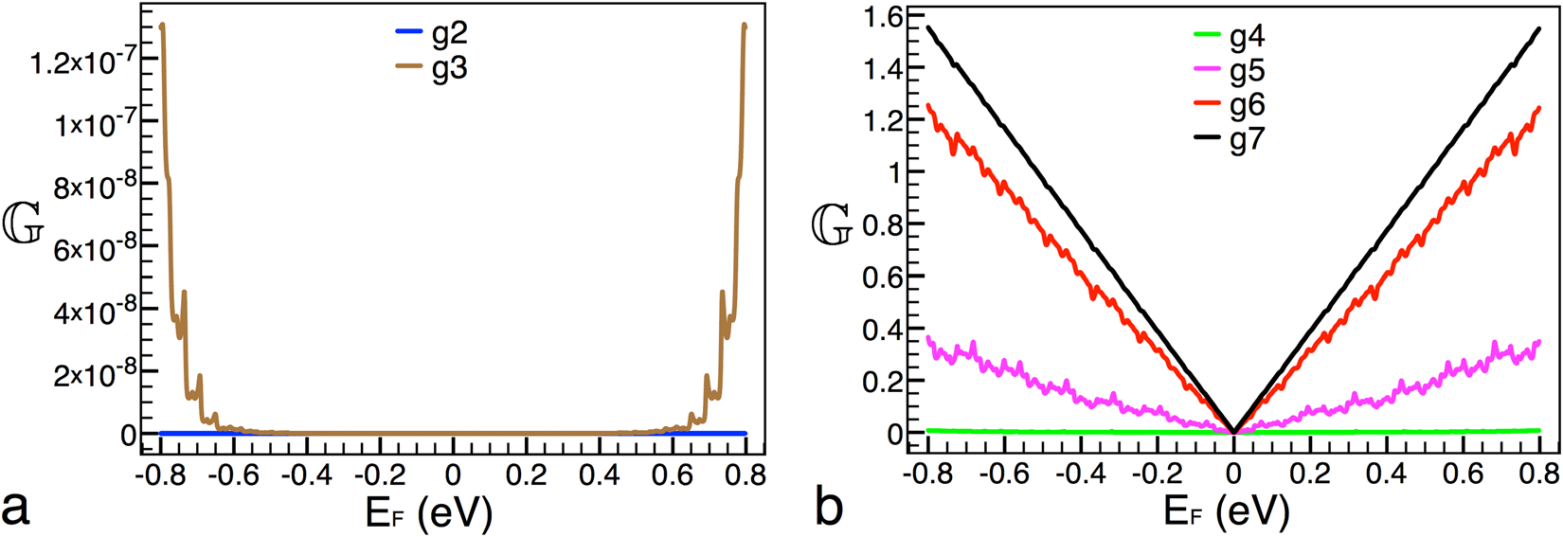
Conductance versus Fermi energy for (**a**) low and (**b**) high generations. The black, red, magenta, green, brown and blue curves correspond to generations 7, 6, 5, 4, 3, and 2, respectively. The structural parameters of the system are the same as in Fig. 2. Tow aspects need to be highlighted: 1) as the generation increases the conductance increases as well, as far as we know this is a general trend in Cantor-like structures; 2) at low generations the conductance patterns are far from being self-similar and generations greater than g7 cannot be consider because the feature size of the system will surpass the Fermi wave length and the carbon-carbon distance in graphene.

**Figure 9:**
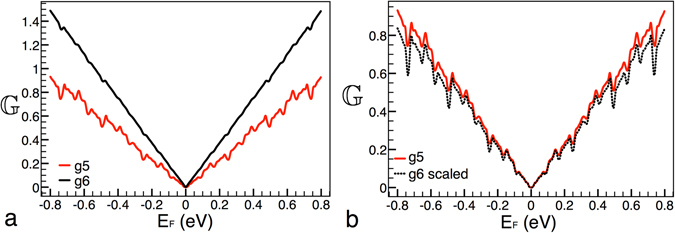
Scaling between generations as in the case of Fig. 2. Here, we are showing that by adjusting the height of the main barrier it is possible to obtain self-similar conductance patterns at lower generations. The non-scaled and scale curves correspond to generations 5 and 6. The height of the main barrier and the length of the system are 0.1 eV and 10000 Å, respectively.

Other aspect that we want to address is the validity of the scaling rules. Regarding this point, it is important to take into account that our system has limits. For instance, at low generations our system is far from being self-similar, then we do not expect that the conductance patterns display self-similarity, see Fig. 8a. Likewise, at high generations the feature size of our system will surpass the carbon-carbon distance, thus our results would be unphysical. For the parameters that we are using the maximum generation that we can reach is g7. Certainly the feature size will reach the Fermi wave length after a few generations, however it is enough to get generations for which the conductance displays self-similarity. For instance, the conductance of generations 5, 6 and 7 manifests self-similar characteristics (Fig. 8b) and the feature size for these generations is in the range of the Fermi wave length in graphene. Even more, we can adjust the height of barriers and the system’s length to reach higher generations with self-similar properties. Our system is also limited by the physical description that we are adopting. For instance, the height of the main barrier cannot be more than 2 eV because the relativistic description is valid up to there, and the length of the system cannot be arbitrarily large because coherent quantum transport will be not longer acceptable. So, these limits define a set of parameters for which conductance patterns will display self-similarity and hence eqs (14)–(17) will hold for this set of parameters. According to the parameters that we use and those that we were able to explore, a minimum of five generations is required to obtain and possibly experimentally observe self-similar conductance patterns, see Fig. 9.

In relation to this point, we also want to mention that the derivation of the scaling rules from a deductive standpoint represent an open problem. As far as we know this is a tough task and challenge mathematically speaking. For instance, even when we use the scaling rule for the transmittance between generations^19^,

$${T}_{g}(E,\theta )={[{T}_{g+m}(E,\theta )]}^{{9}^{m}}$$

it is not possible to obtain the scaling for the conductance because the integral that defines it,

$${\mathbb{G}}={E}_{F}^{\ast }{\int }_{-\frac{\pi }{2}}^{\frac{\pi }{2}}{[{T}_{g+m}({E}_{F}^{\ast },\theta )]}^{{9}^{m}}\,\cos \,\theta d\theta ,$$

cannot be analytically computed such that we obtain the expression that we derived numerically, eq. (14). This argumentation is also applicable to the other scaling rules. So, further mathematical studies are needed to discern if the scaling rules can be obtain analytically.

In fourth place, it is interesting to note that as generation increases the conductance increases as well, see Fig. 8. As far as we know this is as general trend in Cantor-like structures. Likewise, as far as we can see the mentioned trend is directly related to the fragmentation of the structure, width and height of barriers, as the generation increases. This shrinking of the width and height of barriers favors propagating states, giving an overall enhancement of the conductance. According to previous studies in this kind of structures^29^ the bound states spectrum is progressively fragmented as the generation rises. However, it is not at all clear that the fragmentation of the energy spectra be responsible of the conductance enhancement. In our system both the fragmentation of the energy spectra and its relation to the conductance improvement represent open problems.

In fifth place, it is worth mentioning that temperature and disorder effects are very important in practically all solid-state systems. To this respect, our structure is not the exception and it is expected that the self-similar characteristics of the conductance patterns be susceptible to the mentioned effects. Specifically, we expect that for some critical temperature and disorder strength the signatures of self-similarity be totally lost. Experimental results in graphene heterojunctions^5^ confirm that quantum coherence phenomena are visible at temperatures as high as 80 K. Then, we expect that the conductance patterns display self-similar characteristics up to these temperatures. Even more, improvements in the growth and fabrication techniques should lead to higher critical temperatures. Certainly, further studies are needed in order to unveil the particularities of these effects.

Finally, we would like to highlight that the transport in fractal (self-similar) media has captured the attention of physicists and mathematicians for more than thirty years. Plenty of mathematical developments and physical outcomes have been reported. Practically all these results have been obtained from a classical perspective. Within this context, the transport of relativistic electrons in self-similar media is unprecedented and graphene as a laboratory at hand offers a unique opportunity to explore and unveil this novel transport. To this respect, the study of transport properties of Dirac electrons in complex structures is still in its infancy. Recently, the quantum transport in Sierpinski carpets has been studied^30^. As far as we know this work and ours constitute the beginning of this exciting field. However, further theoretical and experimental studies are needed in order to unveil the peculiar transport characteristics of Dirac electrons in complex structures. In this endeavor, the two-dimensional nature of graphene and related materials is ideal, since in principle complex structures of Cantor and Sierpinski type could be fabricated.

## Electronic supplementary material

Supplementary info
