# Supplementary material for: Self-similar conductance patterns in graphene Cantor-like structures
Source: Sci Rep. 2017 Apr 4;7:617. doi: 10.1038/s41598-017-00611-z (PMC5428863; doi:10.1038/s41598-017-00611-z)
Supplement: Supplementary file 1 — Supplementary info [file 41598_2017_611_MOESM1_ESM.pdf]

Supplementary Information

Self-similar conductance patterns in graphene Cantor-like Structures

H. García-Cervantes[1], L. M. Gaggero-Sager[2], D. S. Díaz-Guerrero[1], O. Sotolongo-Costa[1] and I. Rodríguez-Vargas[1,3]

[1]*Centro de Investigación en Ciencias, Instituto de Investigaciones en Ciencias Básicas y Aplicadas (IICBA), Universidad Autónoma del Estado de Morelos, Av. Universidad 1001, Col Chamilpa, 62209, Cuernavaca Morelos, México.* [2]*CIICAp, IICBA, Universidad Autónoma del Estado de Morelos, Av. Universidad 1001, Col. Chamilpa, 62209 Cuernavaca, Morelos, México.* [3]*Unidad Académica de Física, Universidad Autónoma de Zacatecas, Calzada Solidaridad Esquina Con Paseo La Bufa S/N, 98060 Zacatecas, Zac., México.*

S1. Transmittance scaling rules as a function of the energy for oblique incidence

One of the main characteristics of the complex structure under study is that the transmittance scaling rules previously obtained at normal incidence[1] are also valid for oblique incidence, as in the case of simpler Cantor-like structures[2]. In short, the scaling rule between generations comes as,

$$T_G(E,\theta_i) \approx [T_{G+m}(E,\theta_i)]^{9^m}, \tag{S1.1}$$

where G is the generation number, m is the difference between generations and θ_i is the angle of incidence. The results of this expression for a concrete case are shown in Fig. S1. The transmittance scaling between different heights of the main barrier can be written as,

$$T_{V_0}(E,\theta_i) \approx [T_{\frac{V_0}{\kappa}}(E,\theta_i)]^{\kappa^2}, \tag{S1.2}$$

where V_0 is the height of the main barrier and κ is the factor that connects the heights of the barrier. In Fig. S2 we show how this expression works for a specific case. In the case of the scaling between different lengths of the system the expression is,

$$T_{Lt}(E,\theta_i) \approx [T_{\frac{Lt}{\alpha}}(\frac{E}{\alpha},\theta_i)]^{\alpha^2}, \tag{S1.3}$$

with Lt the length of the system and α the scaled factor between the lengths. The results of this expression for a specific case are shown in Fig. S3.

We can combine the individual scaling rules to obtain a general expression,

$$T_{(G,V_0,Lt)}(E,\theta) \approx [T_{(G+m,\frac{V_0}{\kappa},\frac{Lt}{\alpha})}(\frac{E}{\alpha},\theta_i)]^{9^m\cdot\kappa^2\cdot\alpha^2}. \tag{S1.4}$$

In Fig. S4 we show how this expression works. As we can notice the matching between the reference curve (solid-red) and the scaled one (dotted-black) is quite good, see Fig. S4b.

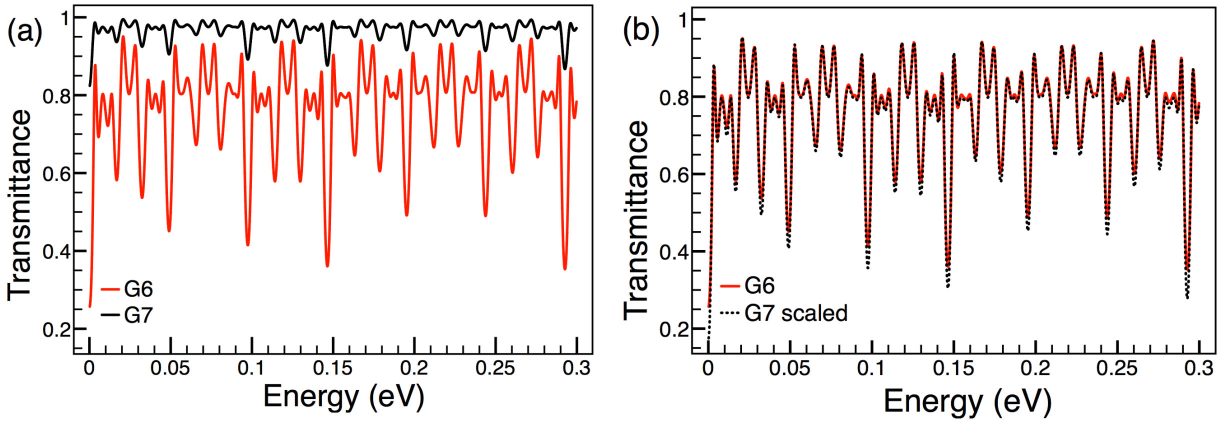

Fig. S1: Transmittance scaling between generations at oblique incidence $\theta_i = \pi/3$. (a) Transmittance patterns for generations 6 (G6) and 7 (G7), solid-red and solid-black lines, respectively. (b) The same as in (a), but in this case generation G7 is scaled according to eq. (S1.1), dotted-black curve. The other parameters of the Cantor-like structures are: $V_0 = 0.2$ eV and $Lt = 10000$ Å.

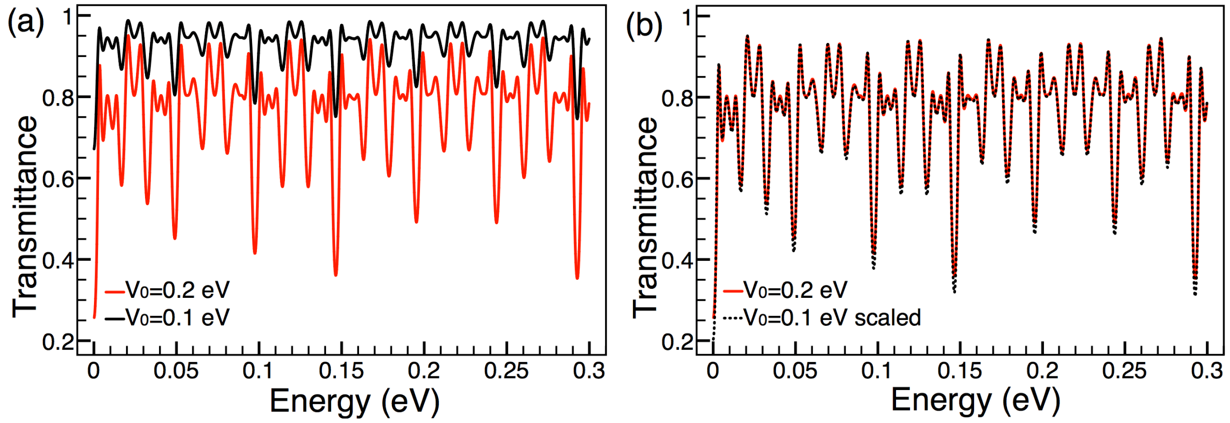

Fig. S2: Transmittance scaling between different heights of the main barrier at oblique incidence $\theta_i = \pi/3$. (a) Transmittance patterns for heights $V_0 = 0.2$ eV and $V_0 = 0.1$ eV, solid-red and solid-black lines, respectively. (b) The same as in (a), but in this case the curve that corresponds to $V_0 = 0.1$ eV is scaled according to eq. (S1.2), dotted-black curve. The other parameters of the Cantor-like structures are: $G6$ and $Lt = 10000$ Å.

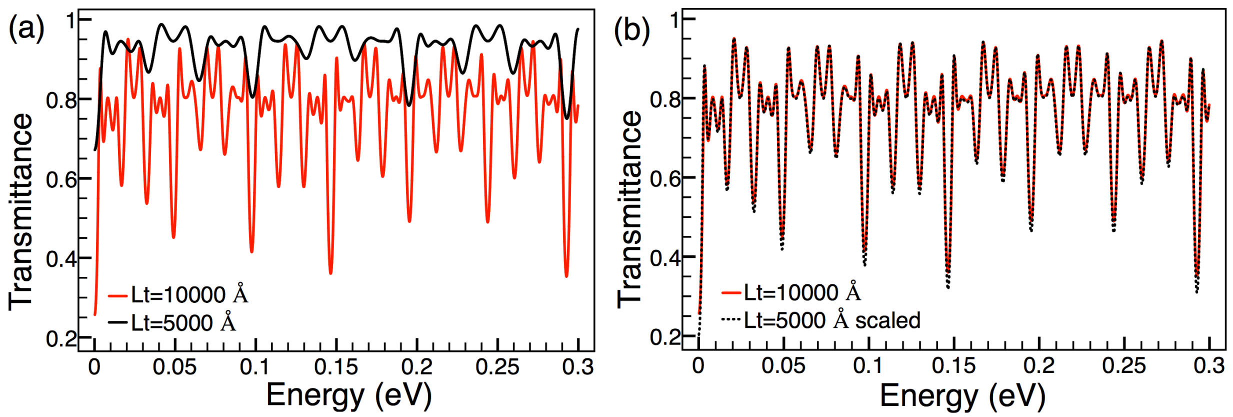

Fig. S3: Transmittance scaling between different lengths of the system at oblique incidence $\theta_i = \pi/3$. (a) Transmittance patterns for lengths $Lt = 10000$ Å and $Lt = 5000$ Å, solid-red and solid-black lines, respectively. (b) The same as in (a), but in this case the curve that corresponds to $Lt = 5000$ Å is scaled according to eq. (S1.3), dotted-black curve. The other parameters of the Cantor-like structures are: $G6$ and $V_0 = 0.2$ eV.

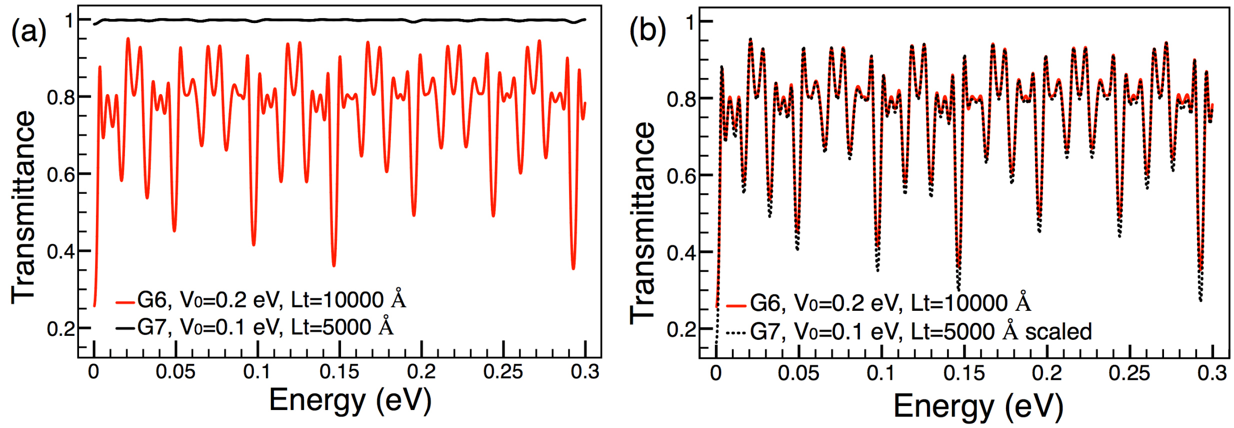

Fig. S4: General transmittance scaling at oblique incidence $\theta_i = \pi/3$: combination of the individual scaling rules. (a) Transmittance patterns for Cantor-like structures with parameters $G6$, $V_0 = 0.2$ eV and $Lt = 10000$ Å and $G7$, $V_0 = 0.1$ eV and $Lt = 5000$ Å, solid-red and solid-black lines, respectively. (b) The same as in (a), but in this case the solid-black curve is scaled according to eq. (S1.4), dotted-black curve.

S2. Transmittance scaling rules as a function of the angle for fixed energy

Other peculiar characteristic of our system is that the self-similar patterns are also present for the angular distribution of the transmittance, that is, the transmittance as a function of the angle for fixed energy. Even more striking is that the scaling rules are practically the same as in the preceding section. The major difference comes in the scaling between lengths of the system, in which it is necessary to re-scale the quantity that we fixed, in this case the energy. As far as we have studied it is fundamental that both relevant coordinates of our system, energy and distance, be scaled in Cantor-like fashion in order to obtain these peculiar transmission properties. In the rest of this section we will show the scaling rules and its results for concrete cases.

The transmittance scaling between generations can be written as,

$$T_G(\theta, E_i) \approx [T_{G+m}(\theta, E_i)]^{9^m}, \tag{S2.1}$$

where E_i is the energy of the incident electrons, quantity that we maintain fixed. The other symbols have the same meaning as in the preceding section. In Fig. S5 we show the results of this scaling rule. As we can see the reference curve and the scaled one match quite well, Fig. S5b. Likewise, the transmission probabilities of systems with different heights of the main barrier, for the same generation and length, can be connected by the following formula,

$$T_{V_0}(\theta, E_i) \approx [T_{\frac{V_0}{\kappa}}(\theta, E_i)]^{\kappa^2}, \tag{S2.2}$$

here κ has the same meaning as in the case of eq. (S1.2). The outcomes of this expression are shown in Fig. S6. In systems with different length, with the same generation and height of the main barrier, the transmittance patterns present self-similar characteristics. The scaling rule that allows us to go from one pattern to the other comes as,

$$T_{Lt}(\theta, E_i) \approx [T_{\frac{Lt}{\alpha}}(\theta, \alpha E_i)]^{\alpha^2}, \tag{S2.3}$$

here E_i needs to be scaled as αE_i, while the variable quantity θ remains unaltered. This expression contrasts with the corresponding one at oblique incidence, eq. (S1.3), in which the variable quantity E needs to be scaled as E/α and the fixed quantity θ_i remains the same. In Fig. S7 we show the results of this expression for a system with lengths 10000 Å and 5000 Å. As we can notice the expression works quite well, see Fig. S7b.

We can combine the individual scaling rules to obtain a general one,

$$T_{(G,V_0,Lt)}(\theta, E_i) \approx [T_{(G+m,\frac{V_0}{\kappa},\frac{Lt}{\alpha})}(\theta, \alpha E_i)]^{9^m\cdot\kappa^2\cdot\alpha^2}. \tag{S2.4}$$

The results of this expression are shown in Fig. S8.

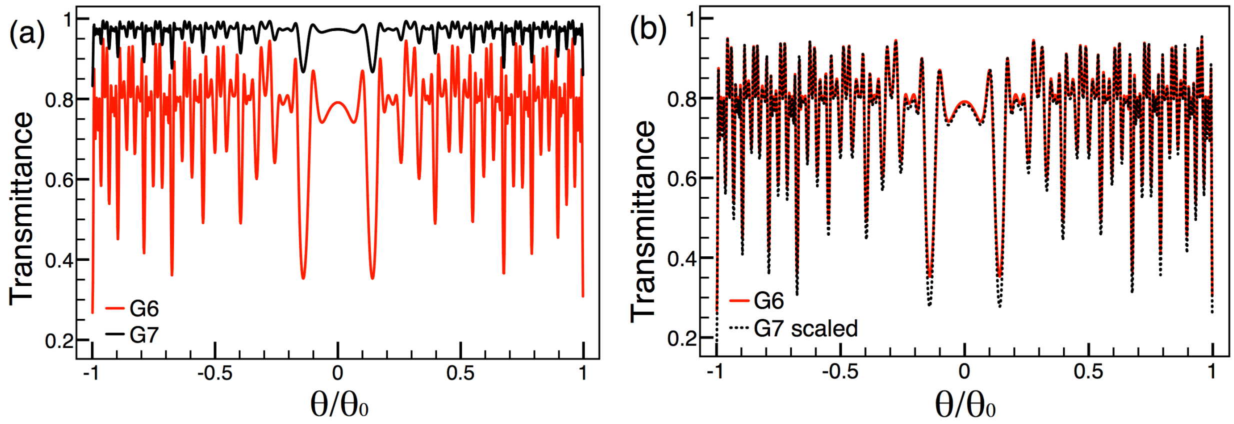

Fig. S5: Transmittance scaling between generations as a function of the angle of incidence. The energy of the incident electrons is fixed at $E_i = 0.15$ eV. (a) Transmittance patterns for generations G6 and G7, solid-red and solid-black lines, respectively. (b) The same as in (a), but in this case generation G7 is scaled according to eq. (S2.1), dotted-black curve. The other parameters of the Cantor-like structure are: $V_0 = 0.2$ eV and $Lt = 10000$ Å.

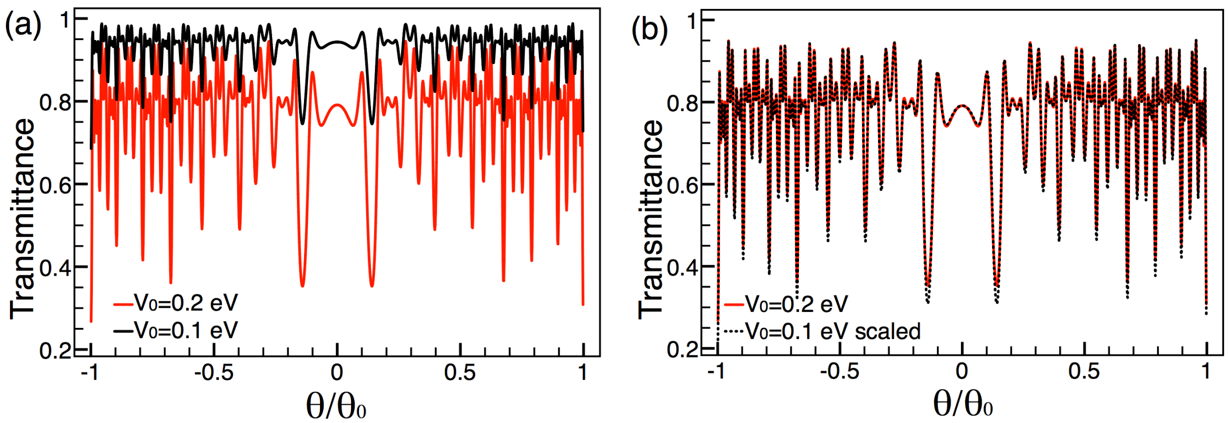

Fig. S6: Transmittance scaling between different heights of the main barrier as a function of the angle of incidence. The energy of the incident electrons is fixed at $E_i = 0.15$ eV. (a) Transmittance patterns for heights $V_0 = 0.2$ eV and $V_0 = 0.1$ eV, solid-red and solid-black lines, respectively. (b) The same as in (a), but in this case the curve that corresponds to $V_0 = 0.1$ eV is scaled according to eq. (S2.2), dotted-black curve. The other parameters of the Cantor-like structure are: $G6$ and $Lt = 10000$ Å.

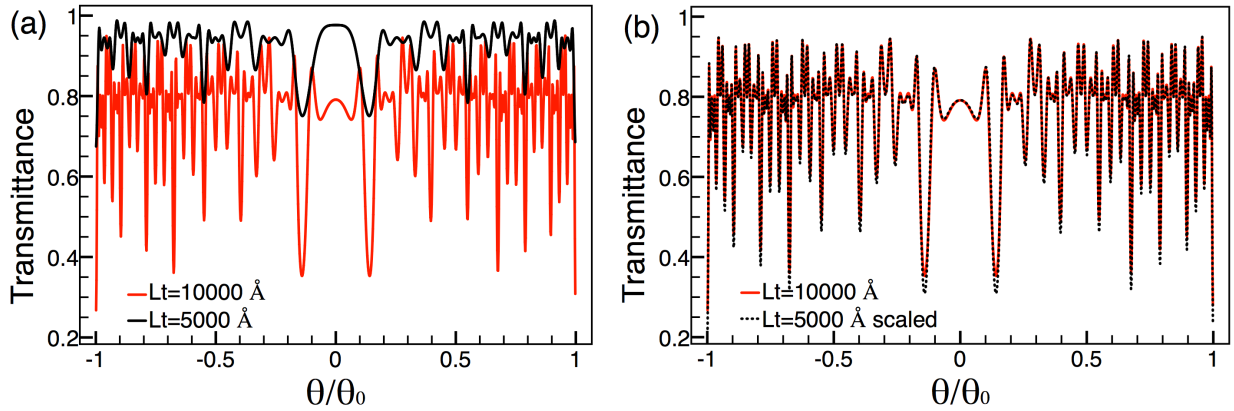

Fig. S7: Transmittance scaling between different lengths of the system as a function of the angle of incidence. The energy of the incident electrons is fixed at $E_i = 0.15$ eV. (a) Transmittance patterns for lengths $Lt = 10000$ Å and $Lt = 5000$ Å, solid-red and solid-black lines, respectively. (b) The same as in (a), but in this case the curve that corresponds to $Lt = 5000$ Å is scaled according to eq. (S2.3), dotted-black curve. The other parameters of the Cantor-like structures are: $G6$ and $V_0 = 0.2$ eV.

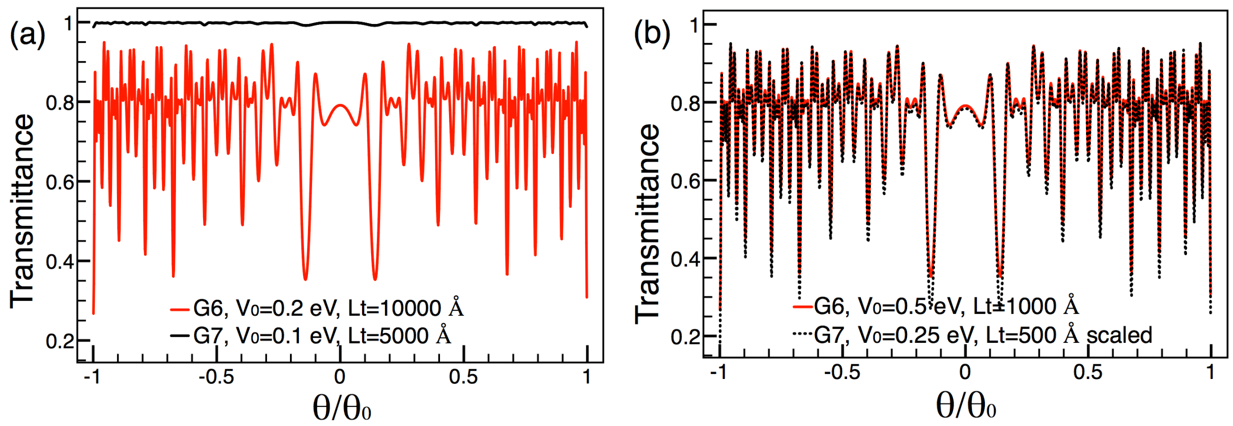

Fig. S8: General transmittance scaling as a function of the angle of incidence: combination of the individual scaling rules. The energy of the incident electrons is fixed at $E_i = 0.15$ eV. (a) Transmittance patterns for Cantor-like structures with parameters $G6$, $V_0 = 0.2$ eV and $Lt = 10000$ Å and $G7$, $V_0 = 0.1$ eV and $Lt = 5000$ Å, solid-red and solid-black lines, respectively. (b) The same as in (a), but in this case the solid-black curve is scaled according to eq. (S2.4), dotted-black curve.

S3. H scaling rules

The results of conductance are always welcomed because it is an experimentally measurable quantity. Then, in principle our findings can be corroborated experimentally. However, conductance is not a suitable quantity to derive the scaling rules because it is not a probabilistic quantity, that is, it is not bounded between 0 and 1. Then, when we test scale factors to relate the conductance patterns the curve that we are scaling grows up rapidly and the matching between the reference and scaled curve is practically impossible. In this sense, the search for the scaling rules becomes cumbersome. Fortunately, we can define a quantity related to the conductance and bounded between 0 and 1. We will name this quantity as H, which will be the angular average of the transmittance over half of the Fermi surface. Formally, H is defined as,

$$\mathbb{H} = \frac{H}{H_0} = \frac{1}{2}\int_{-\frac{\pi}{2}}^{\frac{\pi}{2}} T(E_F^*, \theta)\cos\theta d\theta, \tag{S3.1}$$

where $E_F^* = E_F/E_0$ is the Fermi energy normalized to the height of the barrier $E_0 = V_0 = t'$, $H_0 = \frac{e^2 L_y E_F}{\hbar^2 v_F}$ is the fundamental factor of this re-defined conductance H, L_y is the width of the system in the y coordinate, and θ is the angle of incidence of the electrons with respect to the x coordinate. If we take into account that the conductance is the angular average of the transmission probability over the Fermi surface multiply by the Fermi energy, then we can write G in terms of H as,

$$\frac{G}{G_0} = 2E_F^* \frac{H}{H_0}, \tag{S3.2}$$

or simply

$$\mathbb{G} = 2E_F^* \mathbb{H}, \tag{S3.3}$$

where $G_0 = 2H_0$ is the fundamental factor of the conductance. So, once we obtain the scaling rules for $\mathbb{H}$ the ones for $\mathbb{G}$ are automatically determined via eq. (S3.3). In the rest of this section we will show the scaling rules for $\mathbb{H}$ and its results for specific cases.

For sake of simplicity, we will proceed in the mentioned order, first the scaling rules and then results. However, we have to keep in mind that actually the process to discern the scaling rules is in reverse order, that is, we first obtain results for concrete cases, then we try to connect the $\mathbb{H}$ patterns by scaling the corresponding curves with appropriate factors, and finally we extend the obtained scaling for arbitrary generations, heights of the main barrier and lengths of the system. In short, the $\mathbb{H}$ scaling between generations comes as,

$$\mathbb{H}_G(E_F^*) = \left[\mathbb{H}_{G+m}(E_F^*)\right]^{9^m}, \tag{S3.4}$$

The results for generations $G6$ and $G7$ are shown in Fig. S9. The $\mathbb{H}$ scaling between heights of the main barrier is given as,

$$\mathbb{H}_{V_0}(E_F^*) = \left[\mathbb{H}_{\frac{1}{\kappa}V_0}\left(\frac{E_F^*}{\kappa}\right)\right]^{\kappa^2}. \tag{S3.5}$$

The results for $V_0 = 0.2$ eV and $V_0 = 0.1$ eV are depicted in Fig. S10. The $\mathbb{H}$ scaling between lengths of the system can be written as,

$$\mathbb{H}_{Lt}(E_F^*) = \left[\mathbb{H}_{\frac{1}{\alpha}Lt}\left(\frac{E_F^*}{\alpha}\right)\right]^{\alpha^2}. \tag{S3.6}$$

The outcomes for $Lt = 10000$ Å and $Lt = 5000$ Å are presented in Fig. S11. The general expression that combines all scaling rules is given as,

$$\mathbb{H}_{(G,V_0,Lt)}(E_F^*) = \left[\mathbb{H}_{\left(G+m,\frac{1}{\kappa}V_0,\frac{1}{\alpha}Lt\right)}\left(\frac{1}{\kappa\cdot\alpha}E_F^*\right)\right]^{9^m\cdot\kappa^2\cdot\alpha^2}. \tag{S3.7}$$

Fig. S12 shows the results of this general scaling for particular cases. Finally, we want to stress that it is striking that $\mathbb{H}$ holds well-defined scaling rules. In principle, we cannot expect this because $\mathbb{H}$ is the sum of the dispersion channels. Even more striking is that rules be practically the same as in the case of the transmittance. The major difference comes in the scaling between heights of the main barrier, in which the Fermi energy needs to be re-scaled.

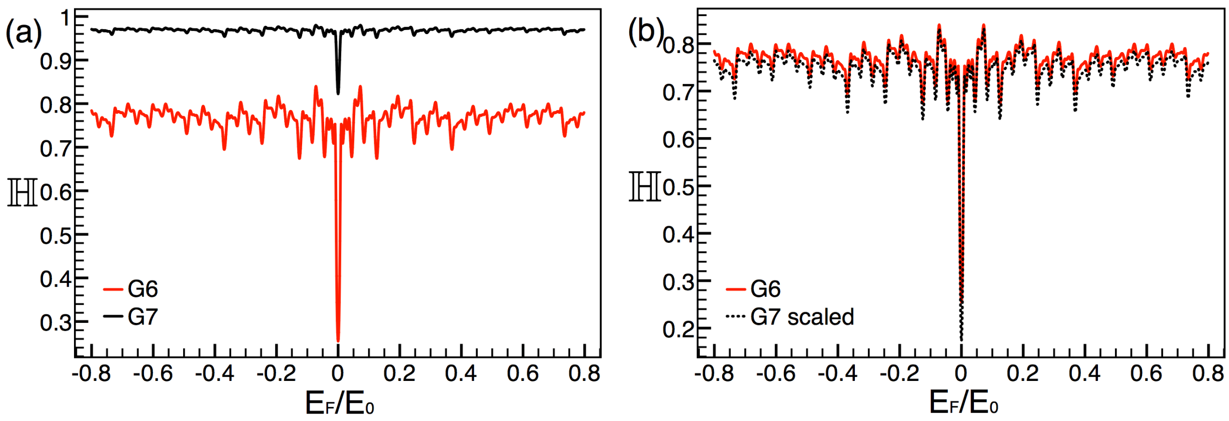

Fig. S9: $\mathbb{H}$ scaling between generations. (a) $\mathbb{H}$ patterns for generations G6 and G7, solid-red and solid-black lines, respectively. (b) The same as in (a), but in this case generation G7 is scaled according to eq. (S3.4), dotted-black curve. The other parameters of the Cantor-like structure are: $V_0 = 0.2$ eV and $Lt = 10000$ Å.

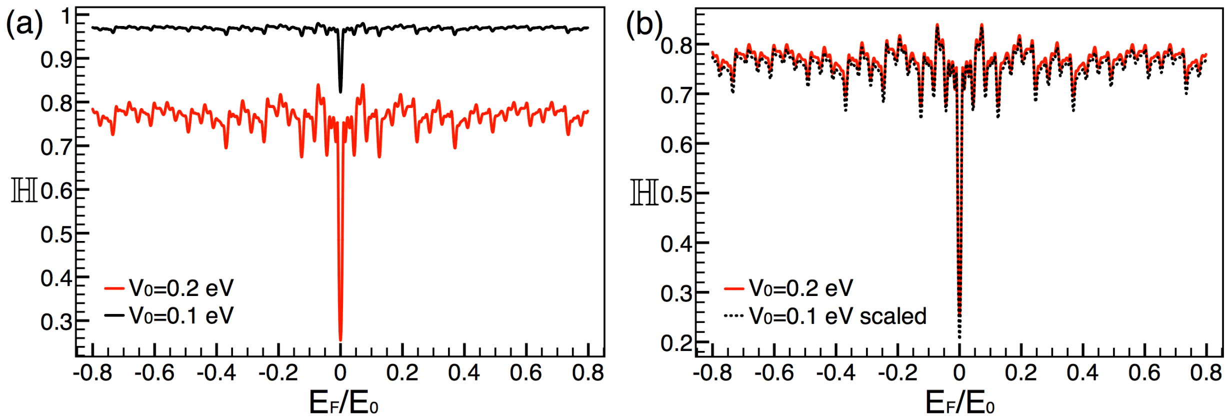

Fig. S10: $\mathbb{H}$ scaling between different heights of the main barrier. (a) $\mathbb{H}$ patterns for heights $V_0 = 0.2$ eV and $V_0 = 0.1$ eV, solid-red and solid-black lines, respectively. (b) The same as in (a), but in this case the curve that corresponds to $V_0 = 0.1$ eV is scaled according to eq. (S3.5), dotted-black curve. The other parameters of the Cantor-like structure are: $G6$ and $Lt = 10000$ Å.

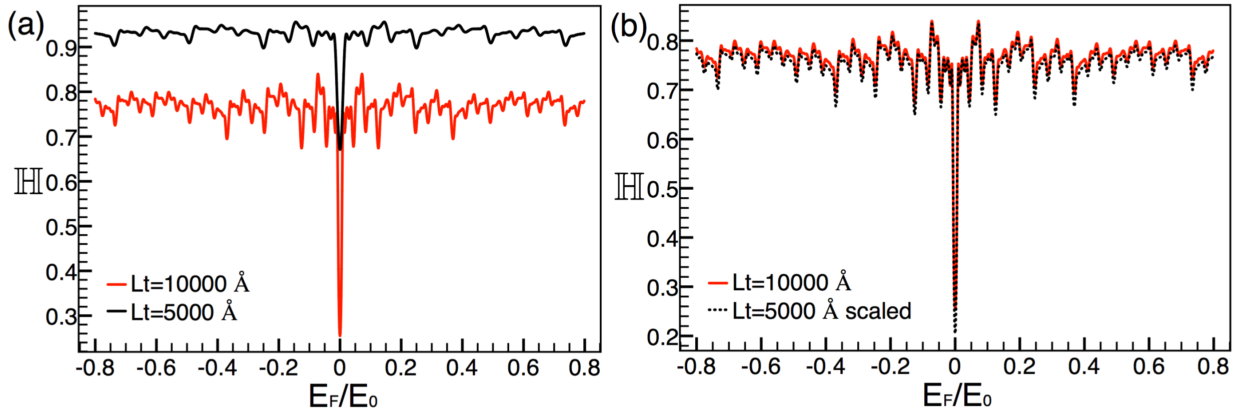

Fig. S11: ℍ scaling between different lengths of the system. (a) ℍ patterns for lengths $Lt = 10000$ Å and $Lt = 5000$ Å, solid-red and solid-black lines, respectively. (b) The same as in (a), but in this case the curve that corresponds to $Lt = 5000$ Å is scaled according to eq. (S3.6), dotted-black curve. The other parameters of the Cantor-like structures are: $G6$ and $V_0 = 0.2$ eV.

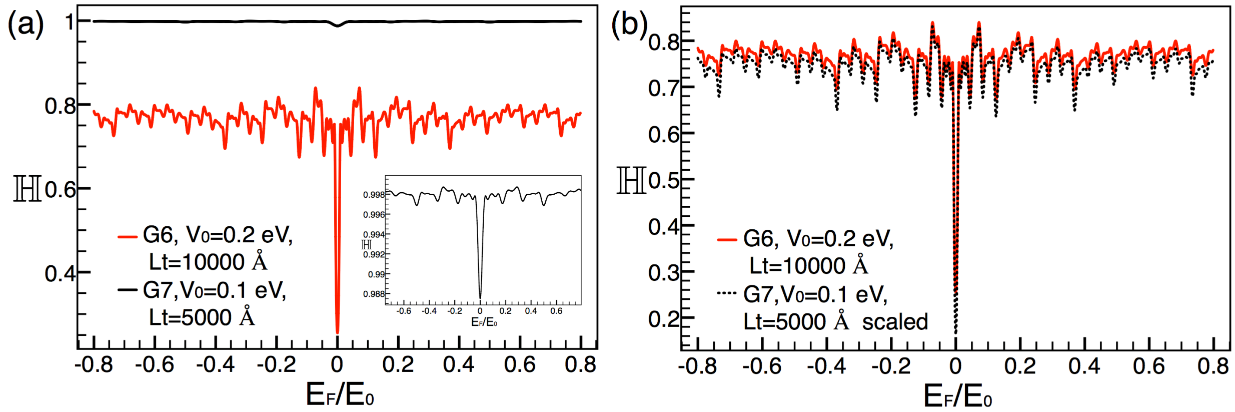

Fig. S12: General ℍ scaling: combination of the individual ℍ scaling rules. (a) ℍ patterns for Cantor-like structures with parameters $G6$, $V_0 = 0.2$ eV and $Lt = 10000$ Å and $G7$, $V_0 = 0.1$ eV and $Lt = 5000$ Å, solid-red and solid-black lines, respectively. The inset shows the ripple structure not seen in the solid-black curve. (b) The same as in (a), but in this case the solid-black curve is scaled according to eq. (S3.7), dotted-black curve.

Supplementary References

1. D.-S. Díaz-Guerrero *et al.*, "Self–similar charge transmission in gapped graphene," Fractals **24**, 1630002 (2016).

2. R. Rodríguez-González *et al.*, "Self-similar transmission properties of aperiodic Cantor potentials in gapped graphene," Eur. Phys. J. B **89**, 17 (2016).
